# Supplementary material for: Quantitative insights into the cyanobacterial cell economy
Source: eLife. 2019 Feb 4;8:e42508. doi: 10.7554/eLife.42508 (PMC6391073; doi:10.7554/eLife.42508)
Supplement: Figure 1—source data 1. [file elife-42508-fig1-data1.pdf]

Ratio of the requirements of selected elements by *Synechocystis* cells and the elements supplemented by the replacement of a spare culture medium during turbidostat experiments in this study.								
**Red light intensity [μE m^{-2} s^{-1}]**	**27.5**	**55**	**110**	**220**	**440**	**660**	**880**	**1100**
**Na**	0.2%	0.4%	0.4%	0.5%	0.4%	0.4%	0.5%	0.5%
**N**	6.1%	9.6%	10.8%	12.9%	9.7%	11.6%	14.4%	12.7%
**Mg**	16.8%	26.4%	29.7%	35.6%	26.7%	31.9%	39.7%	35.1%
**S**	9.4%	14.7%	16.6%	19.9%	14.9%	17.8%	22.2%	19.6%
**Ca**	20.4%	31.9%	36.0%	43.1%	32.4%	38.6%	48.1%	42.5%
**Fe**	40.3%	63.1%	71.2%	85.1%	64.0%	76.4%	95.1%	83.9%
**P**	33.3%	52.2%	58.9%	70.5%	52.9%	63.2%	78.7%	69.5%

Estimated uptake rates of selected elements by *Synechocystis* cells during the turbidostat cultivation [mg L^{-1} h^{-1}]. The calcualtions are based on direct measurements of cellular dry weights and specific growth rates in this study, and on maximal concentration of particular elements in *Synechocystis* biomass as recorded in the literature.								
**Red light intensity [μE m^{-2} s^{-1}]**	**27.5**	**55**	**110**	**220**	**440**	**660**	**880**	**1100**
**Na**	0.03	0.05	0.08	0.14	0.18	0.21	0.20	0.17
**N**	0.42	0.80	1.27	2.23	2.89	3.37	3.18	2.69
**Mg**	0.03	0.07	0.10	0.18	0.24	0.28	0.26	0.22
**S**	0.03	0.05	0.08	0.14	0.18	0.21	0.20	0.17
**Ca**	0.06	0.11	0.17	0.30	0.38	0.45	0.42	0.36
**Fe**	0.01	0.03	0.04	0.07	0.09	0.11	0.10	0.09
**P**	0.05	0.10	0.15	0.27	0.35	0.40	0.38	0.32

Refilling rates of selected elements during *Synechocystis* cultivation in the turbidostat regime, based on data from this study [mg L^{-1} h^{-1}].								
**Red light intensity [μE m^{-2} s^{-1}]**	**27.5**	**55**	**110**	**220**	**440**	**660**	**880**	**1100**
**Na**	11.52	14.08	19.71	28.91	49.90	48.78	36.99	35.45
**N**	6.88	8.41	11.77	17.26	29.80	29.13	22.09	21.17
**Mg**	0.21	0.25	0.35	0.52	0.89	0.87	0.66	0.63
**S**	0.28	0.34	0.48	0.70	1.21	1.18	0.89	0.86
**Ca**	0.27	0.33	0.47	0.69	1.18	1.16	0.88	0.84
**Fe**	0.03	0.04	0.06	0.08	0.14	0.14	0.11	0.10
**P**	0.15	0.18	0.26	0.38	0.65	0.64	0.49	0.47

Weights of selected elements in *Synechocystis* cells, based on directly measured cellular dry weight in this study, and on maximal concentration of particular elements in *Synechocystis* biomass as recorded in the literature [mg L^{-1} h^{-1}].								
**Red light intensity [μE m^{-2} s^{-1}]**	**27.5**	**55**	**110**	**220**	**440**	**660**	**880**	**1100**
**Na**	1.04	1.31	1.37	1.73	1.75	2.05	2.03	1.82
**N**	16.5	20.7	21.6	27.4	27.7	32.5	32.0	28.9
**Mg**	1.36	1.71	1.78	2.26	2.28	2.68	2.64	2.38
**S**	1.03	1.29	1.35	1.71	1.73	2.03	2.00	1.80
**Ca**	2.19	2.75	2.87	3.64	3.67	4.31	4.26	3.83
**Fe**	0.53	0.66	0.69	0.88	0.89	1.04	1.03	0.92
**P**	1.98	2.48	2.59	3.29	3.32	3.90	3.85	3.46

Parameters of *Synechocystis* cultures as measured during the turbidostat experiments in this study.								
**Red light intensity used in this study [μE m^{-2} s^{-1}]**	**27.5**	**55**	**110**	**220**	**440**	**660**	**880**	**1100**
**Specific growth rate μ measured in this study [h^{-1}]**	0.025	0.039	0.059	0.081	0.104	0.104	0.099	0.093
**Flow rate of spare cultivation media measured in this study [h^{-1}]**	0.028	0.034	0.048	0.070	0.121	0.118	0.089	0.086
**Dry weight of *Synecocystis* cells measured in this study [mg L^{-1}]**	132	166	173	220	221	260	256	231

Elemental composition of *Synechocystis* cells based on data available in the literature.					
**Element**	**BG11 [mg L^{-1}]**	**Content in cell [% of DW] Min**	**Max**	**Maximal value recorded in the literature**	**Reference**
**Na**	413.4	0.1	0.8	0.8	Touloupakis et al. 2016
**N**	246.9	8.0	11.2	12.5	Zavřel et al. 2017
		10.0	11.1		Touloupakis et al. 2015
		10.2	11.5		Touloupakis et al. 2016
		11.3	11.3		Shastri and Morgan 2005
		12.5			Kim et al. 2011
		7.1	7.7		Blom 2014
**S**	10.0	0.4	0.8	0.8	Zavřel et al. 2017
		0.4	0.4		Touloupakis et al. 2015
		0.4	0.4		Touloupakis et al. 2016
		0.7			Kim et al. 2011
**Ca**	9.8	0.3	1.7	1.7	Touloupakis et al. 2016
**Mg**	7.4	0.4	1.0	1.0	Touloupakis et al. 2016
**P**	5.4	1.5		1.5	Kim et al. 2011
**Fe**	1.2	0.1	0.4	0.4	Cheng and He 2014
		0.4			Kim et al. 2011

References:

Blom, M., 2014. The Elemental Composition of *Synechocystis* sp . PCC 6803 under Nutrient Limiting Conditions. Bachelor thesis. University of Amsterdam.

Cheng, D., He, Q., 2014. PfsR Is a Key Regulator of Iron Homeostasis in *Synechocystis* PCC 6803. PLoS One 9, 1–13.

Kim, H.W., Vannela, R., Zhou, C., Rittmann, B.E., 2011. Nutrient acquisition and limitation for the photoautotrophic growth of *Synechocystis* sp. PCC6803 as a renewable biomass source. Biotechnol. Bioeng. 108, 277–285.

Shastri, A. a, Morgan, J. a, 2005. Flux balance analysis of photoautotrophic metabolism. Biotechnol. Prog. 21, 1617–26.

Touloupakis, E., Cicchi, B., Benavides, A.M.S., Torzillo, G., 2016. Effect of high pH on growth of *Synechocystis* sp. PCC 6803 cultures and their contamination by golden algae *(Poterioochromonas* sp.). Appl. Microbiol. Biote

Touloupakis, E., Cicchi, B., Torzillo, G., 2015. A bioenergetic assessment of photosynthetic growth of *Synechocystis* sp. PCC 6803 in continuous cultures. Biotechnol. Biofuels 8, 133.

Zavřel, T., Očenášová, P., Červený, J., 2017. Phenotypic characterization of *Synechocystis* sp. PCC 6803 substrains reveals differences in sensitivity to abiotic stress. PLoS One 12, e0189130.
