# Supplementary material for: Quantitative insights into the cyanobacterial cell economy
Source: eLife. 2019 Feb 4;8:e42508. doi: 10.7554/eLife.42508 (PMC6391073; doi:10.7554/eLife.42508)
Supplement: Supplementary file 1. [file elife-42508-supp1.pdf]

parameter	definition	value	source
P_m	cell membrane permeability to inorganic carbon	0.108 [$dm\ h^{-1}$]	(2)
A_{cell}	cell surface area	$1.26 \cdot 10^{-9}$ [$dm^2\ cell^{-1}$]	This study
V_{cell}	cell volume	$4.19 \cdot 10^{-15}$ [$dm^3\ cell^{-1}$]	This study
N_A	Avogadro constant	$6.022 \cdot 10^{23}$ [mol^{-1}]	
k^t_{cat}	maximal import rate	43560 [h^{-1}]	(3)
K_t	half-saturation constant of the transporter enzyme	15 [μM]	(4)
k^m_{cat}	maximal metabolic rate	32700 [h^{-1}]	(5)
K_m	half-saturation constant of the metabolic enzyme	2441560 [molecules $cell^{-1}$]	(5)
γ_{max}	maximal translation rate	79200 [aa h^{-1} $molecules^{-1}$]	(6)
K_a, K_e	half-saturation constant of amino acids and energy units for each reaction	10000 [molecules $cell^{-1}$]	(1)
d_p	protein half-life	1/23 [h^{-1}]	(7)
σ	effective absorption cross-section of the photosynthetic unit	0.7 [nm^2]	This study
τ	maximal turnover rate of the photosynthetic unit	270000 [h^{-1}]	This study
k_d	rate constant for photodamdage	10^{-6}	This study
m_v	energy maintenance rate	$7 \cdot 10^{9}$ [molecules $cell^{-1}$ h^{-1}]	(8)
D_c	average cell density (protein mass per cell)	$1.4 \cdot 10^{10}$ [aa $cell^{-1}$]	(1)
n_R	ribosome length	7358 [aa $molecule^{-1}$]	(1)
n_Q	average protein length for house-keeping proteins	300 [aa $molecule^{-1}$]	This study
n_P	length of one photosynthetic unit	95451 [aa $molecule^{-1}$]	(1)
n_T	transporter length	1681 [aa $molecule^{-1}$]	(1)
n_M	length of one metabolic enzyme complex	28630 [aa $molecule^{-1}$]	(1)
m_α	amount of energy units consumed to create one amino acid	45	(1)
m_c	average carbon chain length of an amino acid	5	(1)
m_γ	amount of energy units needed for one translational elongation step	3	(1)
m_Φ	amount of energy units produced during photosynthesis	8	(1)

Proteome Allocation Problem

$$\max_{\beta,\, X,\, \mu} \quad \mu$$

$$s.t.\ \frac{d[X]}{dt} - \mu \cdot X = 0\ ,$$

$$\sum_j \beta_j = 1\ ,\ \ \forall j \in \mathbb{E}:\ \beta_j \geq 0\ ,$$

$$\sum_j n_j \cdot [j] + [aa] + \frac{[c_i]}{m_c} = D_c\ ,$$

$$n_Q \cdot [Q] = 0.5 \cdot D_c\ ,$$

$$\mathbb{E} = \{R, Q, P, T, M\}\ ,$$

$$X = [ci, aa, e, Q, P^o, P^*, T, M, R]^T \in \mathbb{R}_+\ .$$

ODE System

$$\frac{d[c_i]}{dt} = v_d + v_t - m_c \cdot v_m\ ,$$

$$\frac{d[aa]}{dt} = v_m + n_P \cdot v_i - \sum_j n_j \cdot \gamma_j + d_p \cdot \sum_j n_j \cdot [j]\ ,$$

$$\frac{d[z]}{dt} = \gamma_z - d_P \cdot [z]\ ,$$

$$\frac{d[P^o]}{dt} = \gamma_P - v_1 + v_2 - d_p \cdot [P^o]\ ,$$

$$\frac{d[P^*]}{dt} = v_1 - v_2 - v_i - d_p \cdot [P^*]\ ,$$

$$\frac{d[e]}{dt} = m_\phi \cdot v_2 - v_t - m_\mu \cdot v_m - m_\gamma \cdot \sum_j n_j \cdot \gamma_j - \frac{m_v \cdot [e]}{10 + [e]}\ ,$$

$$\forall j \in \mathbb{E}\ , \forall z \in \mathbb{E} \backslash P\ .$$

Reaction Rates

$$v_d = P_m \cdot \frac{A_{cell}}{V_{cell}} \cdot (N_A \cdot V_{cell} \cdot [c_i^x] - [c_i])\ ,$$

$$v_t = [T] \cdot k^t_{cat} \cdot \frac{[c_i^x]}{K_t + [c_i^x]} \cdot \frac{[e]}{K_e + [e]}\ ,$$

$$v_m = [M] \cdot k^m_{cat} \cdot \frac{[c_i]}{K_m + [c_i]} \cdot \frac{[e]}{K_e + [e]}\ ,$$

$$\gamma_j = [R] \cdot \beta_j \cdot \frac{\gamma_{max}}{n_j} \cdot \frac{[aa]}{K_a + [aa]} \cdot \frac{[e]}{K_e + [e]}\ ,$$

$$v_1 = \sigma \cdot light \cdot [P^o]\ ,$$

$$v_2 = \tau \cdot [P^*]\ ,$$

$$v_i = k_d \cdot \sigma \cdot light \cdot [P^*]\ ,$$

$$\forall j \in \mathbb{E}\ .$$

REFERENCES

1. Faizi M, Zavrel T, Loureiro C, Cerveny J, Steuer R (2018). A model of optimal protein allocation during phototrophic growth. Biosystems 166: 26-36.
2. Mangan NM, Brenner MP (2014). Systems analysis of the CO2 concentrating mechanism in cyanobacteria. eLife 3: p. e02043.
3. Dornmair K, Overath P, Jähnig F (1989). Fast measurement of galactoside transport by lactose permease. J Biol Chem 26(1): 342-346.
4. Omata T, Takahashi Y, Yamaguchi O, Nishimura T (2002). Structure, function and regulation of the cyanobacterial high-affinity bicarbonate transporter, BCT1. Funct Plant Biol 29(3): 151-159.
5. Marcus Y, Altman-Gueta H, Finkler A, Gurevitz M (2005). Mutagenesis at two distinct phosphate-binding sites unravels their differential roles in regulation of Rubisco activation and catalysis. J Bacteriol 187 (12): 222-4228.
6. Bremer H, Dennis P (2008). Modulation of chemical composition and other parameters of the cell at Dif- ferent Exponential Growth Rates. EcoSal Plus, 3(1).
7. Maier T, Schmidt A, Güell M, Kühner S, Gavin AC, Aebersold R, Serrano L (2011). Quantification of mRNA and protein and integration with protein turnover in a bacterium. Mol Syst Biol 7: 511.
8. Knoop H, Gründel M, Zilliges Y, Lehmann R, Hoffmann S, Lockau W, Steuer R (2013). Flux balance analysis of cyanobacterial metabolism: the metabolic network of Synechocystis sp. PCC 6803. PLoS Comput Biol 9(6): e1003081.
