# Supplementary material for: Quantitative insights into the cyanobacterial cell economy
Source: eLife. 2019 Feb 4;8:e42508. doi: 10.7554/eLife.42508 (PMC6391073; doi:10.7554/eLife.42508)
Supplement: Transparent reporting form [file elife-42508-transrepform.docx]

***eLife’s* transparent reporting form**

We encourage authors to provide detailed information *within their submission* to facilitate the interpretation and replication of experiments. Authors can upload supporting documentation to indicate the use of appropriate reporting guidelines for health-related research (see [EQUATOR Network](http://www.equator-network.org/%20)), life science research (see the [BioSharing Information Resource](https://biosharing.org/)), or the [ARRIVE guidelines](http://www.plosbiology.org/article/info:doi/10.1371/journal.pbio.1000412) for reporting work involving animal research. Where applicable, authors should refer to any relevant reporting standards documents in this form.

If you have any questions, please consult our Journal Policies and/or contact us: [editorial@elifesciences.org](mailto:editorial@elifesciences.org).

**Sample-size estimation**

- You should state whether an appropriate sample size was computed when the study was being designed
- You should state the statistical method of sample size computation and any required assumptions
- If no explicit power analysis was used, you should describe how you decided what sample (replicate) size (number) to use

Please outline where this information can be found within the submission (e.g., sections or figure legends), or explain why this information doesn’t apply to your submission:

The number of biological replicates per each analysis is stated in the caption of Figure 2, Figure 2-source data 1 (cellular physiology/morphology; 3-11 biological replicates), in the caption of Figure 3, in Figure 3A and Figure 3-source data 1 (proteomics; 5 biological replicates), in Figure 6-source data 1 and Figure 6-Figure supplement 1 (immunoblotting; 5 biological replicates), as well as within the section Results.

No explicit statistical analysis of samples size estimation was applied prior the experiments since the study is purely microbiological and each sample already represents an average of a population of millions of individual cells.

The usual practice of measuring at least 3 independently cultivated microbial populations was exceeded by carrying the experiments in 5 independent bioreactors and by providing 5 or more biological replicates wherever possible (as described in section Results: Establishing a controlled and reproducible cultivation setup, as well as in Figure 3A).

**Replicates**

- You should report how often each experiment was performed
- You should include a definition of biological versus technical replication
- The data obtained should be provided and sufficient information should be provided to indicate the number of independent biological and/or technical replicates
- If you encountered any outliers, you should describe how these were handled
- Criteria for exclusion/inclusion of data should be clearly stated
- High-throughput sequence data should be uploaded before submission, with a private link for reviewers provided (these are available from both GEO and ArrayExpress)

Please outline where this information can be found within the submission (e.g., sections or figure legends), or explain why this information doesn’t apply to your submission:

All samples were collected within the period of three months. This is not stated in the manuscript since we do not consider this information relevant for the purpose of the study, especially in the context of highly reproducible growth of the experimental strain throughout four successive years of 2013 – 2017, as shown in Figure 1 and as described in section Results: Establishing a controlled and reproducible cultivation setup.

The number of biological replicates per each analysis is stated in the caption of Figure 2, Figure 2-source data 1 (cellular physiology/morphology; 3-11 biological replicates), in the caption of Figure 3, in Figure 3A and Figure 3-source data 1 (proteomics; 5 biological replicates), in Figure 6-source data 1 and Figure 6-Figure supplement 1 (immunoblotting; 5 biological replicates), as well as within the section Results.

No outliers were encountered.

Proteomics data have been deposited to the ProteomeXchange Consortium under accession code PXD009626, as stated in section Material and Methods: Analytical methods: Peptide and protein identification.

**Statistical reporting**

- Statistical analysis methods should be described and justified
- Raw data should be presented in figures whenever informative to do so (typically when N per group is less than 10)
- For each experiment, you should identify the statistical tests used, exact values of N, definitions of center, methods of multiple test correction, and dispersion and precision measures (e.g., mean, median, SD, SEM, confidence intervals; and, for the major substantive results, a measure of effect size (e.g., Pearson's r, Cohen's d)
- Report exact p-values wherever possible alongside the summary statistics and 95% confidence intervals. These should be reported for all key questions and not only when the p-value is less than 0.05.

Please outline where this information can be found within the submission (e.g., sections or figure legends), or explain why this information doesn’t apply to your submission:

All statistical analyses are described in section Materials and Methods: Statistical analysis.

(For large datasets, or papers with a very large number of statistical tests, you may upload a single table file with tests, Ns, etc., with reference to sections in the manuscript.)

**Group allocation**

- Indicate how samples were allocated into experimental groups (in the case of clinical studies, please specify allocation to treatment method); if randomization was used, please also state if restricted randomization was applied
- Indicate if masking was used during group allocation, data collection and/or data analysis

Please outline where this information can be found within the submission (e.g., sections or figure legends), or explain why this information doesn’t apply to your submission:

The samples were allocated into individual experimental groups according to light intensity under which the cyanobacteria were cultivated. As indicated in section Results: Establishing a controlled and reproducible cultivation setup, light intensity was the only variable in the study.

No masking was used during group allocation, data collection and/or data analysis.

**Additional data files (“source data”)**

- We encourage you to upload relevant additional data files, such as numerical data that are represented as a graph in a figure, or as a summary table
- Where provided, these should be in the most useful format, and they can be uploaded as “Source data” files linked to a main figure or table
- Include model definition files including the full list of parameters used
- Include code used for data analysis (e.g., R, MatLab)
- Avoid stating that data files are “available upon request”

Please indicate the figures or tables for which source data files have been provided:

There are source data available for Figures 1, 2, 3 and 6 and Tables 1 and 2. Additionally, all figures contain Figure supplements, including model definition file (Figure 5-Figure supplement 1). Proteomics data have been deposited to the ProteomeXchange Consortium under accession code PXD009626, as stated in section Material and Methods: Analytical methods: Peptide and protein identification.
